# Political ideology shapes support for the use of AI in policy-making

**DOI:** 10.3389/frai.2024.1447171

**Published:** 2024-10-30

**Authors:** Tamar Gur, Boaz Hameiri, Yossi Maaravi

**Affiliations:** ^1^Adelson School of Entrepreneurship, Reichman University, Herzliya, Israel; ^2^The School of Social and Policy Studies, Tel Aviv University, Tel Aviv, Israel

**Keywords:** artificial intelligence (AI), political ideology, artificial intelligence in governance, technology acceptance, governance

## Abstract

In a world grappling with technological advancements, the concept of Artificial Intelligence (AI) in governance is becoming increasingly realistic. While some may find this possibility incredibly alluring, others may see it as dystopian. Society must account for these varied opinions when implementing new technologies or regulating and limiting them. This study (*N* = 703) explored Leftists’ (liberals) and Rightists’ (conservatives) support for using AI in governance decision-making amidst an unprecedented political crisis that washed through Israel shortly after the proclamation of the government’s intentions to initiate reform. Results indicate that Leftists are more favorable toward AI in governance. While legitimacy is tied to support for using AI in governance among both, Rightists’ acceptance is also tied to perceived norms, whereas Leftists’ approval is linked to perceived utility, political efficacy, and warmth. Understanding these ideological differences is crucial, both theoretically and for practical policy formulation regarding AI’s integration into governance.

## Introduction

“There can be no serious conflicts on Earth, in which one group or another can seize more power than it has for what it thinks is its own good despite the harm to Mankind as a whole, while the Machines rule.” (Isaac Asimov, I, Robot)

In this quote, Asimov argues that the destructive human tendency to hoard power and resources for one’s group while harming others may be curbed by (artificially intelligent, i.e., AI) robotic guidance. This idea of AI-guided governing was completely unrealistic until recently. However, AI is gradually finding its way into every aspect of our lives, including our governments (Chiusi et al., 2020; Coglianese and Ben Dor, 2020; Makridakis, 2017). AI is currently used to decipher medical tests (Yu et al., 2018), and various research papers include analyses conducted by AI and even parts that AI engines like Chat GPT wrote (see Böhm et al., 2023; Alser and Waisberg, 2023; Garg et al., 2023). AI has been increasingly integrated into legal systems that, in many states, suffer from great delays due to case overload (Sharma et al., 2022). Moreover, some countries have already piloted or incorporated AI in various branches of governance. For example, in Poland, AI is forming recommendations regarding resource allocations to maximize employment. Although these are meant to be revised by a person, the responsible clerks question less than 1% of recommendations (Kuziemski and Misuraca, 2020). This raises the question: Who will support this innovation, embracing the idea of an artificially intelligent, non-human decision-maker? Also, under what conditions would people be willing to waver the idea of self-governing in favor of AI governance?

### The rise of generative AI

We are currently in the course of an ongoing technological revolution. Retailers use AI to estimate demand, evaluate market potential for new products, and analyze consumers’ online responses (Mariani and Wamba, 2020). AI is also used to predict medical patients’ risks and initiate personalized medicine on a large scale (Israel Innovation Authority, 2018). Generative Artificial Intelligence (GAI) such as ChatGPT, Claude, Bard, and others are now available to the public and used by an increasing number of people for diverse purposes such as education (Baidoo-Anu and Ansah, 2023), medicine (Shoja et al., 2023), mental health care (Tal et al., 2023), as well as scientific analyses and writing (Alser and Waisberg, 2023; Garg et al., 2023). Yet, another field AI is gradually entering is public service and governance (Sousa et al., 2019). Incorporating AI in governance has an immense potential to better our lives and advance our society. Governments could save funds by automating work, using AI to reduce “red tape” and improve the services given to the public (Aung et al., 2021; Golding and Nicola, 2019; van Noordt and Misuraca, 2022). For example, AI has been increasingly integrated into legal systems, where it promises more efficient and fairer outcomes through automation (Sharma et al., 2022). Also, Canada has been incorporating AI into its public services, enhancing the efficiency and effectiveness of its governmental operations. This includes using AI to assist with government forms and respond to public inquiries, streamlining traditionally more time-consuming and labor-intensive processes. Additionally, Canada has piloted an AI immigration decision-making system that evaluates immigration applications, provides recommendations, and notes potential red flags in applications (Kuziemski and Misuraca, 2020).

However, like any technological revolution, it has great disruptive and destructive potential. Experts’ concerns regarding the adverse effects of using AI technologies range from privacy and copyright issues to fear of global annihilation (Bartneck et al., 2021; Hristov, 2016; Yudkowsky, 2008; Yudkowsky et al., 2010). More moderate assessments of the concerns regarding the use of AI refer to issues of fairness, ethical use, inconsistency in service delivery, and discrimination (Nzobonimpa and Savard, 2023). For instance, AI often relies on datasets that recount past information unvetted for fairness or representativeness. Consequently, the data guiding AI’s current decision-making might be biased, reflecting past inequalities rather than ideal, equitable norms (Caliskan et al., 2017). Furthermore, AI technology is essentially different from biological intelligence and other technologies, and we have no clear understanding of how it may further develop and interact with the world (Yudkowsky, 2008). Therefore, we cannot deduce or even have any basis for estimating the implications of the AI revolution on society (Yudkowsky et al., 2010).

As with any new technology, some people rush to use AI technologies, while others lag (Lockey et al., 2021; Sharma et al., 2021). Knowing who is more likely to adopt these new technologies and what contexts may lead to increased use of them is crucial for the mitigation of their negative repercussions as well as reaping their benefits. Past research found that acceptance of AI-based decision-making systems is associated with peoples’ expectations of the system’s performance, the anticipated effort that using them would require, social influence (such as perceived group norms), and having the needed resources to use them (Sharma et al., 2022). People’s preference to use or avoid using AI may also be affected by characteristics of the person, like world views (belief in equality and collectivism) as well as income, gender (being male), irreligiosity, education, and familiarity with AI. These were all found to be associated with the acceptance of AI systems decision-making (Mantello et al., 2023).

### Political ideology

One aspect that has yet to be thoroughly investigated and is likely to impact how individuals perceive AI and their willingness to endorse its application in governance is political ideology. Political ideology is a set of attitudes that encompasses cognitive, emotional, and motivational aspects. Political ideology organizes peoples’ views of the world and their values and helps to explain their political behavior (Tedin, 1987). Political ideology is therefore associated not only with voting behaviors but with how a person sees the world and their personality. Rightists (i.e., conservatives) and Leftists (i.e., liberals) tend to prioritize different values. Rightists generally favor binding values that promote social order and cohesion within communities and groups, while Leftists typically emphasize individuating values focused on individual rights, personal freedoms, and social justice (Stewart and Morris, 2021). Consequently, Rightists are often more influenced by values such as tradition, loyalty to the group, and respect for authority, whereas Leftists are generally more motivated by values such as inclusiveness, personal freedoms, and social justice (Haidt and Graham, 2007).

According to the Uncertainty-Threat Model of political conservatism, Rightists (i.e., conservatives) perceive the world as exceedingly threatening and feel a great need to avoid uncertainty. Therefore, they tend to resist change in general and social change specifically and be more accepting of social inequality since it provides a known structure and stability (Jost et al., 2003a, 2003b). Indeed, studies found that political right-wing ideology is associated with less preference for social equality and a reduced tendency to support marginalized groups (Kluegel, 1990; Skitka and Tetlock, 1993). Further findings indicate that Leftists tend to be curious and seek innovations, while Rightists are conventional and more organized (Carney et al., 2008). When examining the associations between ideology and The Big Five personality traits, Leftists (i.e., liberals) were found to be more open to new experiences (Jost et al., 2003a, 2007), while Rightists show greater conscientiousness (Van Hiel et al., 2004).

These differences should translate to different perceptions and levels of support for using AI in governance decisions. While Rightists may be more deterred by its unconventionality and the uncertainty it brings, Leftists may find these aspects less intimidating and be more intrigued by the innovation that AI may present. Indeed, recent studies found that Rightists showed less support for technological innovations due to binding value concerns (Claudy et al., 2024). However, the context in which a decision or evaluation is made may also influence the ways in which Rightists and Leftists view the use of technological advances. For instance, one research found that Rightists favored government use of automated decision systems such as AIs more than Leftists (Schiff et al., 2022). However, another research found that Leftists prefer support for the use of AI in policing is higher than Rightists and that the support of each varies for different contextual factors such as local sheriffs versus FBI use of AI and use for internal review of officers versus predictive policing of the public (Schiff et al., 2023).

### Trust in the government and acceptance of AI

Many contextual-situational factors were found to increase (or decrease) trust in AI, thus affecting support for integrating AI into governance (Kaplan et al., 2023; Madhavan and Wiegmann, 2007). For instance, people have less trust in AI regarding decisions and tasks that are typically human (intuitive judgments, emotional responses, etc.) but more trust in AI regarding seemingly logical decisions and tasks (Lee, 2018; Stai et al., 2020). People tend to have greater support for AI making medical resource allocation decisions after being exposed to information emphasizing racial and economic inequality in medical outcomes (Bigman et al., 2021). Similarly, having more trust in the system is associated with greater trust in a human physician than an AI; however, less trust in the medical system is associated with no such preference for a human over an AI (Lee and Rich, 2021). Furthermore, people from countries that are characterized by high levels of corruption tend to support the use of AI in decision-making more than people from countries that are not characterized by corruption (Castelo, 2023).

In recent years, there has been a surge of right-wing organizations and political parties disseminating messages aimed at eroding trust in governing institutions and challenging their legitimacy on a global scale (Greven, 2016; Rodrik, 2021). For instance, a recent survey conducted in the US found that about 4 in 10 Leftists (Democrats) and 7 in 10 Rightists (Republicans) do not trust the federal government to do what is right (Hitlin and Shutava, 2022).^1^ This phenomenon can weaken democratic systems and may impact the extent to which individuals trust AI in shaping governance decisions. Although individuals tend to place greater trust in human judgment regarding decisions involving elements of intuition, morality, or emotions (Lee, 2018); when trust in the existing system is notably diminished, people may begin to place AI-generated decisions on par with those made by individuals who represent the system (Lee and Rich, 2021).

## The current research

Similar to numerous other nations, Israel has experienced substantial political turmoil in recent times. Israelis were called to vote in national elections five times between 2019 and 2022. The government that was established following the 2022 November elections was the most Right-winged in Israel’s history,^2^ and soon after its formation, it set out to reform Israel’s governing systems. The suggested reform would allow the government greater power while weakening the judicial system, similarly to changes that steered Poland and Hungary away from liberal democracy (Gidron, 2023). At the time (as is still true at the time these lines are being written), the fate of Israel’s democracy seems unclear.

In the past, it was suggested that there is a gap in our understanding of peoples’ attitudes regarding the use of AI indicating that additional research is needed to explore the influence of political ideology on support for the use of AI in governance in various contexts (Schiff et al., 2022). This study was conducted during the initial waves of protests that washed through Israel shortly after the proclamation of the government’s intentions to initiate reform.^3^ In this study, we examined the interplay between political ideology and the current political situation in Israel. We hypothesized that Leftists would show greater support for using AI in governance decision-making since they tend to be more open to changes and seek innovations (Carney et al., 2008). Interestingly, in the case of AI use, the Leftist tendency to embrace innovation may be somewhat contradictory with Leftist values since AI uses past decisions to make current decisions, which may lead to both biased and conservative patterns (Buolamwini and Gebru, 2018; Chouldechova, 2017; Eubanks, 2017). In the context of this research, Leftists may be more concerned that the new reform could increase inequality and social injustice and, therefore, be motivated to limit its power, even by an unknown mechanism such as AI. This is somewhat like Schiff et al. (2023) suggestion that Leftists may support the use of AI in policing not because they believe it is unbiased but because they believe that the current police bias is worse. We did not phrase assumptions regarding the different features associated with support for using AI in governance decision-making for Leftists versus Rightists.

## Methods

The institutional ethics committee approved the research.^4^ The comprehensive study plan, items (translated to English), data, and code required for replicating the results presented in this study can be accessed through the Open Science Framework at the following link: https://osf.io/sbdjc/?view_only=d2ddcf4f920b41209e1b45f4a99d3b4a. This study was not preregistered.

### Sample

A representative sample in terms of gender, age, and place of residence of Jewish Israeli society (*N* = 703; 53.6% women; *M*age = 48.74, *SD* = 16.52) was recruited via an online survey company (HaMidgam).^5^ Regarding political ideology, 43.1% of the participants defined themselves as Rightists, 32.7% as centrists, and 24.2% as Leftists.^6^ The checkmarket sample size calculator^7^ was used to assess the minimal required sample size for the population of 7.106 million Jews (Israel Central Bureau of Statistics, 2022). A 4% margin of error and a 95% confidence interval yielded a minimal sample size of 601 participants. The required sample size of 650 participants was calculated using G*Power version 3.1.9.4 (Faul et al., 2007; Faul et al., 2009). This calculation was based on conducting a multiple linear regression analysis involving 29 predictors. The anticipated effect size was set at *f^2^* = 0.07, which falls between a small and medium effect and the desired statistical power of 0.8. These specifications yielded a desired sample size of 325 participants. However, the analysis was planned to be carried out separately for two groups (Rightists and Center-Leftists); thus, the total sample size needed was doubled, resulting in a target of 650 participants.

### Procedure

Data was collected by a survey company (“Midgam Project Web Panel”), between April 3rd and April 5th, 2023. Participants were asked to participate in exchange for monetary compensation, provided informed consent, and proceeded to complete the study questionnaire. Participants reported their attitudes and perceptions regarding AI and decision-making by the government at the time. Because there were fewer participants with left-leaning political views in our sample, we merged the center and left-leaning individuals into a single “Center-Leftist” group (56.9%) for comparison with the “Rightist” group (43.1%).

### Measures

As this study was exploratory, we investigated a range of measures. To effectively categorize these measures, we have separated them into two groups: those related to AI and technology and those concerning government and the suggested reform.

#### Measures related to technology and AI

*Technology knowledge (Tech Know)*. Participants’ perception of their technological literacy was assessed using participants’ responses to 4 items adapted from Mantello et al. (2023), e.g., “How familiar are you with coding and programming?” (𝛼 = 0.86). High scores denote high perceived self-knowledge of technology. The scales were all translated to Hebrew, and unless otherwise mentioned, responses to all scales ranged from 1 (completely disagree) to 6 (greatly agree). *Note:* This and the following variables will appear in an abbreviated form in Table. The abbreviated forms of the variables are listed in brackets next to their entire label.

*Technology readiness (Tech readiness)*. Willingness to accept and use new technologies, in general, was assessed via participants’ responses to 18 items previously used by Lam et al. (2008), e.g., “You enjoy the challenge of figuring out high tech gadgets” (𝛼 = 0.86). High scores denote willingness to adopt new technologies.

*Legitimacy of using AI in governance (Legitimacy^(AI)^)*. Perceived legitimacy was assessed using five items regarding fairness, equality, and safety. Equality items were adapted from the legitimacy scale used by Starke and Lünich (2020), e.g., “The AI-based governance decision-making process would be fair” (𝛼 = 0.85). High scores denote high perceived legitimacy of the use of AI in governance decision making.

*Usefulness of AI in governance (Usefulness^(AI)^)*. The perceived usefulness of AI was assessed by six items adapted from Maaravi and Heller (2021), e.g., “Using AI-based decision-making mechanisms may improve the effectiveness of governance management” (𝛼 = 0.80). High scores denote high perceived efficiency (effectiveness) of the use of AI in governance decision making.^8^

*Warmth toward AI (Warmth^(AI)^)*. We assessed warmth toward AI using the feeling Thermometer. The feeling thermometer is a prevalent measure of general positivity versus negativity toward an entity often used to assess affective polarization (Gidron et al., 2019). Participants rated their feelings toward AI on a thermometer scale ranging from 0 (highly cold/unfavorable) to 100 (highly warm/favorable).

*Emotions regarding using AI in governance*. Participants were asked to assess their own emotional state when thinking of AI being used to make governance decisions. Participants rated how much fear, anxiety, excitement, despair, anger, hope, and sadness they felt at using AI in governance decision-making.

*Fear of personal harm if AI is used in governance (Fear of harm^(AI)^)*. Perceived risk of harm was assessed by three items, e.g., “Are you concerned that decisions made using AI could harm your or your family members’ well-being? “(𝛼 = 0.89). High scores denote perceived high risk or high levels of fear for oneself and one’s family due to use of AI in governance decision making.

*Perceived public support regarding the use of AI in governance (Norms^(AI)^)*. Perceived norms were examined using two items in which the participant assessed the percentage of Israelis supporting using AI in governance decision-making. The items were: “In your opinion, what percentage of Israelis support the use of AI as a tool for governance decision-making?” and “In your opinion, what percentage of Israelis believe that governance decisions made by AI are legitimate?.” Participants responded to these items using a slider ranging from 0 to 100% (*r* = 0.74, *p* < 0.001). High scores denote the perception that the use of AI in governance is normative and supported by much of society. ^9^

*Attitudes regarding AI use in governance (Attitudes^(AI)^)*. Attitudes regarding the use of AI as an aid in governance decisions were assessed using two items adapted from Maaravi and Heller (2021), e.g., “Using AI-based decision-making mechanisms as a tool in governance management is a good idea” (*r* = 0.76, *p* < 0.001). High scores denote the acceptance of the use of AI in governance decision-making.^10^

*Support for the use of AI in governance decision-making (Support^(AI)^).* Support for the use of AI was assessed by eight items adapted from Maaravi and Heller (2021), e.g., “I will support the use of AI as often as possible in governance decision-making” (𝛼 = 0.89). High scores denote support for the use of AI in governance decision-making.

#### Measures related to the current government

*Political efficacy (Political efficacy)*. Participants’ political self-efficacy (i.e., the perception that one can affect the political processes or situations) was assessed by two items: “As a citizen, you can influence the decision-making processes in the country” and “As a citizen, you can influence policy decisions that will affect your future” (*r* = 0.77, *p* < 0.001). High scores denote high political self-efficacy.

*Warmth to* var*ious factions in the government (Warmth^(GOV)^)*. We assessed warmth toward the government using the feeling Thermometer (Gidron et al., 2019). Participants rated their feelings toward the current government and the coalition on a thermometer scale ranging from 0 (highly cold/unfavorable) to 100 (highly warm/favorable).

*Emotions regarding the current government.* Participants were asked to assess their emotional state when thinking of the current government making governance decisions. Participants rated how much fear, anxiety, excitement, despair, anger, hope, and sadness they felt at the thought regarding the current government’s decision-making. High scores denote feeling more intense emotions.

*Fear of personal harm due to the current government’s political decisions (Fear of harm^(GOV)^)*. Perceived risk of harm was assessed by three items; e.g., “Are you concerned that decisions made by the current government could harm your or your family members’ well-being? “(𝛼 = 0.94). High scores denote perceived high risk or high levels of fear for oneself and one’s family due to the current government political decisions.

*Public support of the reform (Norms^(GOV)^)*. Perceived norms were examined using six items in which the participant assessed the percentage of Israelis supporting the government’s proposed reforms, e.g., “In your opinion, what percentage of Israelis support the government’s latest reforms?.” Participants responded to these items using a slider ranging from 0 to 100% (𝛼 = 0.77). High scores denote the perception that much of society supports the proposed reform.

Participants also reported social demographic measures and other additional measures not used in the current paper (for the complete questionnaire, see Appendix C).

## Results

We first examined the differences between Rightists’ and Center-leftists’ perceptions, attitudes, and emotions regarding the use of AI within governance decision-making. As hypothesized, the results indicate that compared with Rightists, Center-leftists reported more favorable attitudes toward technology in general and specifically toward the use of AI within governance decision-making, more positive emotions and less negative emotions regarding the use of AI within governance, and greater support for the use of AI in governance decision making. Interestingly, Center-leftists and Rightists did not differ in the extent to which they rated their technology knowledge or their views about public support (norms) regarding the use of AI in governance decision-making.

We then examined the differences between Rightists’ and Center-leftists’ perceptions, attitudes, and emotions regarding the government, its governance decision-making, and the proposed reforms. The results indicate that Center-leftists reported more negative attitudes and feelings toward the government, its governance decision-making, and the proposed reforms than Rightists. Interestingly, Rightists and Center-leftists did not differ in their perceived political efficacy (despite having a right-wing government; See Table 1).

**Table 1:** Means, standard deviations (in parentheses), and comparisons between rightists and center-leftists.

	Center-left	Right	*t*		Center-left	Right	*t*
Fear of harm^(AI)^	2.94 (1.19)	3.32 (1.25)	−4.02*	Fear of harm^(GOV)^	4.58 (1.26)	3.42 (1.56)	10.63*
Norms^(AI)^	38.04 (18.71)	38.25 (18.39)	−0.15	Norms^(GOV)^	34.33 (12.81)	45.42 (17.02)	−9.49*
Warmth^(AI)^	58.24 (23.96)	49.85 (25.33)	4.49*	Warmth^(GOV)^	11.59 (18.61)	48.76 (35.10)	−16.74*
Sadness^(AI)^	2.16 (1.31)	2.28 (1.36)	−1.21	Sadness^(GOV I)^	4.70 (1.47)	3.01 (1.73)	13.66*
Fear^(AI)^	2.80 (1.30)	2.93 (1.43)	−1.25	Fear^(GOV)^	4.60 (1.47)	3.03 (1.70)	12.83*
Anxiety^(AI)^	2.73 (1.34)	2.86 (1.44)	−1.24	Anxiety^(GOV)^	4.63 (1.50)	3.07 (1.74)	12.46*
Excitement^(AI)^	3.13 (1.37)	2.79 (1.37)	3.28*	Excitement^(GOV)^	1.69 (1.11)	2.58 (1.49)	−8.74*
Despair^(AI)^	2.07 (1.25)	2.27 (1.40)	−1.95	Despair^(GOV)^	4.74 (1.42)	2.99 (1.74)	14.20*
Anger^(AI)^	2.02 (1.25)	2.32 (1.40)	−2.92	Anger^(GOV)^	4.73 (1.44)	3.04 (1.72)	13.82*
Hope^(AI)^	3.28 (1.29)	3.04 (1.30)	2.38	Hope^(GOV)^	1.59 (0.99)	2.99 (1.56)	−13.69*
Tech Know	2.82 (1.14)	2.77 (1.08)	0.54	Political efficacy	2.70 (1.07)	2.78 (1.23)	−0.93
Tech readiness	4.14 (0.72)	3.98 (0.68)	2.94				
Attitudes^(AI)^	3.52 (1.09)	3.36 (1.15)	1.96				
Legitimacy^(AI)^	3.51 (0.94)	3.27 (0.95)	3.39*				
Usefulness^(AI)^	3.78 (0.87)	3.58 (0.90)	3.01				
Support^(AI)^	3.14 (1.01)	2.85 (1.01)	3.76*				

Due to Bonferroni Correction; *p < 0.0018 (0.05/27). Measures that refer to the use of AI are marked with (AI), and measures that refer to the government and its proposed reforms are marked with (GOV).

**Figure 1:**
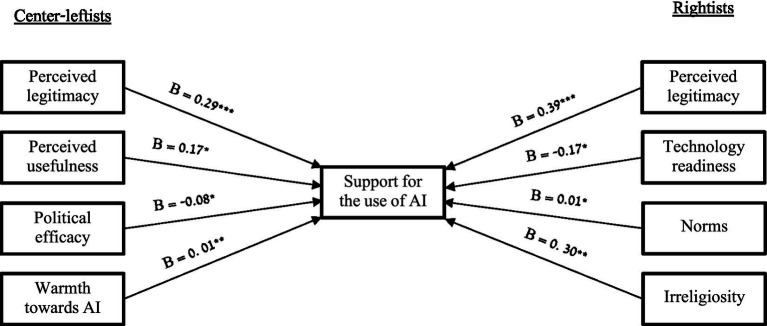
Significant predictors of support for using AI in governance. *p < 0.05; **p < 0.01; ***p < 0.001.

Finally, we conducted an exploratory analysis of the data to examine the issues associated with support for using AI in governance. We conducted two linear regressions (separately for Center-leftists and Rightists) with support for using AI in governance as an outcome variable and the other measures mentioned above as predictors (while holding age and gender). Among the Center-leftists, the significant predictors of support for the use of AI were perceived usefulness of the use of AI (*B =* 0.17, *SE = 0*.08, *t =* 2.04, *p = 0*.042), warmth toward AI (*B =* 0.01, *SE = 0*.002, *t =* 2.85, *p =* 0.005), and political efficacy (more political efficacy associated with less support for the use of AI; *B =* −0.08, *SE =* 0.04, *t =* −2.20, *p =* 0.028). For Rightists, technology readiness (*B = −*0.17, *SE = 0*.08, *t =* −1.98, *p* = 0.049), perceived public support (*B* = 0.01, *SE* = 0.003, *t* = 2.21, *p* = 0.028), and irreligiosity (i.e., being secular; *B =* 0.30, *SE = 0*.10, *t =* 3.04, *p =* 0.003) were significant predictors of support for the use of AI in governance decision-making. Interestingly, when controlling for all other variables, technology readiness predicts less support for using AI in governance. Perceived legitimacy of AI as an aid in making governance decisions predicted support for the use of AI as a governance decision-making tool among both Center-leftists (*B =* 0.29, *SE =* 0.08, *t =* 3.83, *p <* 0.001) and Rightists (*B =* 0.39, *SE =* 0.09, *t =* 4.24, *p <* 0.001; see Appendix B for complete statistics; Figure 1).

## Discussion

This research investigates the relationship between political ideology and new technologies in a particular political context, focusing on support for using AI in governance. The study was conducted in Israel during political unrest, as a radical political reform pushed forward by an extreme Right-wing government faced significant public opposition. It explores how Rightists (conservatives) and Leftists (liberals) differ in their acceptance and perception regarding allowing AI to have a role in governance decision-making. As expected, our findings show that Center-leftists feel greater warmth and excitement regarding the use of AI and are less fearful of using AI in governance while also perceiving it as more legitimate than Rightists.

Our study analyzed the differences between Rightists and Center-leftists in Israel. During the survey period, a Rightist block supported the right-wing government and its reforms, while a similar-sized block of Centrists and Leftists opposed them. Notably, though Leftists were a minority, many Centrists also resisted the government’s policies (Anabi, 2022; Hermann and Anabi, 2023b; Hermann and Anabi, 2023a). Our findings reveal stark contrasts between these groups’ attitudes and emotions regarding the government and its proposed reforms. Center-leftists generally exhibited less warmth, hope, and excitement and heightened despair, anxiety, anger, sadness, and fear, including concerns for personal and family safety and well-being.

The study also reveals different underlying factors between Rightists’ and Center-leftists’ support for using AI in governance. Both groups’ support was influenced by perceived legitimacy. However, Rightists are swayed by social norms regarding AI use, technology readiness, and irreligiosity, while Leftists are influenced by perceived usefulness, political efficacy, and warmth toward AI. Surprisingly, among Rightists, higher levels of technology readiness predicted less support for using AI in governance. A possible explanation for this surprising finding is that the perceived legitimacy of AI and norms regarding the use of AI (and other predictors like the perceived usefulness of AI) account for most of the variance in support for using AI in governance having to do with perceptions of safety, efficiency, and legitimacy. After controlling for these predictors, the remaining variance that technology readiness can account for may have to do with being comfortable with current technologies, while AI may signify a very different type of technology which may be intimidating.

These results may signal that Rightists’ support for using AI in governance is rooted in social acceptance. In contrast, Leftists’ support is rooted in usefulness and the potential for change in situations they find objectionable. This research highlights the complex interplay between political ideology, technology adoption, and current political climates, particularly in contexts like Israel’s precarious political situation.

The findings extend the Uncertainty-Threat Model of political conservatism to a new, previously unstudied context (i.e., the use of AI in governance decision-making). According to the Uncertainty-Threat Model (Jost et al., 2003a), rightists tend to avoid change as it is often ripe with uncertainty that they are very averse to, while the use of AI represents the possibility of immense changes and uncertainty as there is so much that is unknown about this new technology. We also found that one issue that underlies Rightists (but not Leftists) support for using AI was perceived norms, i.e., how accepted by society is this specific use for AI. This is consistent with past research that found that perceived norms influence Rightists’ behavior more than Leftists (Cavazza and Mucchi-Faina, 2008; Kaikati et al., 2017).

Leftists’ perceptions of AI are interesting since, on the one hand, Leftists seem to be more open to societal changes and innovations (Carney et al., 2008; Hibbing et al., 2014), yet AI is an innovation that uses existing societal knowledge to make new decisions and thus may reinforce existing societal structures and discriminatory processes that are inconsistent with liberal values (Howard and Borenstein, 2018; Mehrabi et al., 2021). For instance, due to ad optimization algorithms, women are exposed to fewer advertisements promoting jobs in Science, Technology, Engineering, and Math (STEM) fields (Lambrecht and Tucker, 2019). Also, AI used by courts in the United States was found to falsely evaluate African American offenders’ risk of committing another crime as higher than White offenders (Mehrabi et al., 2021). Our findings indicate that while Leftists were more supportive of the use of AI in making decisions, the concerns that underlie their support were associated less with perceptions of fairness and more with the practicality of the matter, i.e., usefulness and political efficacy (that refers to whether they felt they could change governance in Israel in another way).

The current study adds to the body of knowledge regarding how Leftists and Rightists respond to new technological situations, thus adding to our understanding of the issues that underlie peoples’ support for new technologies and the differences between Leftists and Rightists. This examination focused on the increasing power we give AI mechanisms over our lives, an irrelevant issue until very recently but looms over us as AI mechanisms are integrated into more and more areas of our lives. These findings are important as they expose possible differences regarding the issues that affect support for the use of AI within government decision-making among people with different political ideologies.

### Limitations and future directions

The current study examined different responses based on political orientation during a politically turbulent time in Israel’s history. This led to some interesting findings, yet future studies should examine these issues in other countries and different political situations to disentangle the effects of political orientation from the effects of one’s perception of the government. We examined people’s responses to the use of AI in governance but did not delve into the various branches of government AI could be used in or the different roles it may have. Future research should investigate support for AI use in specific governance functions, thus allowing for more nuanced insights, as different applications of AI might carry different meanings. We compared the responses of Rightists to those of Center-leftists due to the low percentage of Leftists in the current population of Israel. Future studies should examine support for using AI in governance in populations that allow for a representative yet balanced sample of Rightists and Leftists.

We found that perceptions of legitimacy underlie both Rightists and Center-leftists’ support for using AI in governance. However, we did not delve into what that legitimacy might entail, as Rightists and Leftists may stress different aspects of legitimacy. For instance, when referring to fairness, do the Rightists and Leftists note interactional fairness, distributive fairness, or informational fairness; when referring to an appropriate and satisfactory process, do the Rightists and Leftists refer to the same things? Future studies should more thoroughly examine the mechanisms that lead to one’s choice regarding giving AI mechanisms great power. Future studies should also further examine additional spheres in which people may support using AI in making crucial decisions and further examine issues that underlie peoples’ support for using AI in making decisions. Future studies may track changes in attitudes over time, and examine these changes in light of political events and developments in AI technology. This research focused on people’s perceptions of AI and support for using it; another important avenue of research that should be examined in the future is the actual utility and drawbacks of different uses of AI. Future research should identify policy areas where AI could be most beneficial, compare outcomes of AI-driven vs. human-driven policies, and examine strategies to ensure unbiased, democratic AI-driven policies. Finally, additional research is needed to address ethical implications and strategies for maintaining ethics and public trust in AI-driven governance.

## Conclusion

The current research found different issues underlie Leftists and Rightists’ support for using AI in governance. However, beyond the contribution to the understanding of political ideology and support for innovations and the use of AI, this paper points to a broader societal aspect: individuals in dire situations may demonstrate varying levels of willingness to embrace new technologies, while it is not clear whether they fully comprehend or consider the potential consequences. This variability in receptivity underscores the need for thoughtful policy design. Whether the goal is to promote the adoption of emerging technologies or to implement regulatory measures, policymakers and inventors of new technologies must consider the diverse attitudes and circumstances of those affected. Recognizing these nuances will be essential in navigating the intricate landscape of AI integration into governance decision-making processes.

## Data Availability

The data and code used for the statistical analysis presented in this study can be accessed through the Open Science Framework at the following link: https://osf.io/sbdjc/?view_only=d2ddcf4f920b41209e1b45f4a99d3b4a.

## Footnotes

^1^The survey was conducted during Biden’s presidency.

^2^This government included the traditionally moderate right Likud party, ultra-Orthodox parties (i.e., Shas and Yahadut Hatora), and the Religious Zionism alliance of Right-wing parties. Prior to 2022, the Religious Zionism alliance was seen by both moderate Leftists and moderate Rightists as too extreme to be included in the government.

^3^This survey was conducted before the 2023 October 7th attack on Israel and the war that followed.

^4^Approval number 023 (dated April 2, 2023).

^5^For additional demographic information about the sample, see Appendix A.

^6^In this sample, we intentionally overrepresented leftists, who comprise approximately 11% of the population (Anabi, 2022), to ensure a sufficient number for analysis. However, even with the oversampling of Leftists, there was no balance in the percentage of Rightists and Leftists. Therefore, we combined the Leftists and the Centrists to create a Center-Leftist group, which we compared to the Rightist group.

^7^Checkmarket sample size calculator, retrieved from: https://www.checkmarket.com/sample-size-calculator/#sample-size-calculator.

^8^Participants responded to this measure twice, and their response consistency was examined. We found no significant differences in patterns of responses given in each of the times, t (702) = 0.59, *p* = 0.559; *r* = 0.85, *p* < 0.001.

^9^An additional item (What percentage of Israelis do you think believe that using artificial intelligence as a tool for policy decision-making should be avoided altogether?) was removed as it reduced Cronbach’s Alpha reliability.

^10^An additional item (The thought of using AI-based decision-making mechanisms in state management is unpleasant) was removed as it reduced Cronbach’s Alpha reliability.
