# Supplementary material for: Political ideology shapes support for the use of AI in policy-making
Source: Front Artif Intell. 2024 Oct 30;7:1447171. doi: 10.3389/frai.2024.1447171 (PMC11557559; doi:10.3389/frai.2024.1447171)
Supplement: Supplementary file 1 [file Data_Sheet_1.pdf]

Supplementary Materials: Political ideology shapes support for the use of AI in policy-making

Appendix A: Methods

Sample representativeness. The demographic depiction of participants that completed the survey is similar, yet not identical to the traits of the Jewish Israeli general population. See Table 1 for the demographic information of the sample and the population according to the Israel Central Bureau Of Statistics.

Table 1. Demographic depiction of the sample and the Jewish Israeli general population.

		Sample	Population
Gender	Women	53.6	50.4
	Men	46.4	49.6
Age	18-24	6.0	13.8
	25-34	17.2	19.0
	35-44	23.2	18.5
	45-54	14.5	15.0
	55-64	14.4	13.2
	65+	24.8	20.4
District of Residence	Jerusalem District	7.0	11.4
	Northern District	8.5	9.2
	Haifa District	15.1	10.5
	Central District	29.2	28.7
	Tel Aviv District	27.2	19.7
	Southern District	10.2	14.1
	Judea and Samaria Area	2.8	6.4
Place of birth[1]	Israel	78.2	78.7
	Asia and The Middle East	0.7	2.3
	North America and Oceania	1.6	1.7
	South America	1.6	1.0
	Europe (including the former USSR)	17.0	12.2
	Africa	0.9	4.2
Votes 2022 Elections	Likud	15.8	23.4

[1] This depiction relates to 97% of the participants. Additional 21 participants (3%) did not report their place of birth.

	Yesh Atid Party	33.4	17.8
	Religious Zionism Party	6.8	10.8
	National Unity Party	10.2	9.1
	Shas	1.0	8.2
	United Torah Judaism	0.7	5.9
	Yisrael Beitenu	4.6	4.5
	Labor	5.5	3.7
	Meretz	6.3	3.2
	Ra’am	-	4.1
	Hadash-Ta’al	0.1	3.8
	Jewish Home Party	1.3	1.2
	Balad	-	2.9
	Additional small parties	-	1.1
	Did not vote or did not report their vote	8.4	-
Perceived economic status	No income	1.0	-
	Much lower than average	14.6	-
	Below average	20.5	-
	Average	31.8	-
	Above average	14.6	-
	Much higher than average	10.2	-
	Unreported	7.2	-

Information about the general population presented in Table 1 was retrieved from formal sources listed below. Gender, district of residence, and place of birth were retrieved from the Israel Central Bureau Of Statistics (2021). Age distribution in the adult Jewish population was retrieved from the Israel Central Bureau Of Statistics (2020). Voting patterns were retrieved from the **Israel Democracy Institute** (2022). These are the actual election results. As such, it refers to the general population in Israel (not only Jews) and thus may be somewhat different from the sample population (Israeli Jews only). Unfortunately, we were unable to find formal descriptives of perceived economic status.

Appendix B: Additional Results.

We found that Center-leftist participants tended to be less religious compared with Rightists, $\chi^2(3) = 100.48$, $p < .001$. Further tests revealed that among Center-leftists, there was a greater percentage of secular participants, $\chi2(1) = 98.77$, $p < .001$, while among Rightists, there was a greater percentage of each of the non-secular groups (*all p's* < .01).

Table 2. Participants religiosity among Rightists and Center-leftist (absolute numbers)

	Secular	Traditional	Religious	Ultra-orthodox
**Rightists**	169	85	40	9
**Rightists (expected)**	225.8	51.3	13.2	4.7
**Center-leftists**	355	34	9	2
**Center-leftists (expected)**	298.2	67.7	27.9	6.3

We conducted an exploratory analysis of the data to examine the issues associated with support for using AI in governance. We conducted two linear regressions (separately for Center-leftists and Rightists) with support for using AI in governance as an outcome variable and the other measures mentioned in the paper as predictors (while holding age and gender).

Table 3. Predictors of support for the use of AI as an aid in making governance decisions

	Center-Left	Right
**Fear of harm$^{(AI)}$**	$B = -0.02$, $SE = 0.04$, $t = -0.45$, $p = .657$	$B = -0.05$, $SE = 0.05$, $t = -0.94$, $p = .351$
**Norms$^{(AI)}$**	$B = 0.00$, $SE = 0.00$, $t = 0.76$, $p = .445$	$B = 0.01$, $SE = 0.00$, $t = 2.21$, $p = .028$

**Warmth(AI)**	B = 0.01, SE = 0.00, t = 2.85, p = .005	B = 0.00, SE = 0.00, t = 0.25, p = .803
**Sadness(AI)**	B = 0.00, SE = 0.05, t = 0.39, p = .969	B = -0.00, SE = 0.06, t = -0.05, p = .963
**Fear(AI)**	B = -0.10, SE = 0.05, t = -1.88, p = .060	B = -0.03, SE = 0.05, t = -0.64, p = .524
**Anxiety(AI)**	B = 0.03, SE = 0.05, t = 0.54, p = .587	B = -0.09, SE = 0.06, t = -1.59, p = .113
**Excitement(AI)**	B = 0.05, SE = 0.04, t = 1.05, p = .296	B = 0.04, SE = 0.05, t = 0.73, p =.467
**Despair(AI)**	B = 0.04, SE = 0.05, t = 0.71, p = .480	B = 0.05, SE = 0.06, t = 0.80, p = .423
**Anger(AI)**	B = 0.02, SE = 0.05, t = 0.33, p = .742	B =- 0.01, SE = 0.06, t = -0.09, p = .926
**Hope(AI)**	B = 0.10, SE = 0.05, t = 1.88, p = .062	B = 0.10, SE = 0.05, t = 1.90, p = .059
**Tech Know**	B = -0.01, SE = 0.04, t = -0.32, p = .746	B = 0.03, SE = 0.05, t = 0.65, p = .515
**Tech readiness**	B = -0.10, SE = 0.07, t = -1.56, p = .121	B = -0.17, SE = 0.08, t = -1.98, p = .049
**Attitudes(AI)**	B = 0.09, SE = 0.07, t = 1.29, p = .197	B = 0.04, SE = 0.08, t = 0.48, p = .633
**Legitimacy(AI)**	B = 0.29, SE = 0.08, t = 3.83, p = <.001	B = 0.39, SE = 0.09, t = 4.24, p = <.001
**Usefulness(AI)**	B = 0.17, SE = 0.08, t = 2.04, p = .042	B = 0.05, SE = 0.10, t = 0.55, p = .581
**Fear of harm(GOV)**	B = -0.02, SE = 0.04, t = -0.48, p = .631	B = 0.01, SE = 0.05, t = 0.26, p = .796
**Norms(GOV)**	B = 0.00, SE = 0.00, t = -0.01, p = .106	B = -0.00, SE = 0.00, t = -0.53, p = .598
**Warmth(GOV)**	B = 0.00, SE = 0.00, t = -0.67, p = .993	B = 0.00, SE = 0.00, t = 0.34, p = .737
**Sadness(GOV I)**	B = -0.01, SE = 0.06, t = -0.19, p = .849	B = 0.02, SE = 0.06, t = 0.30, p = .764

**Fear(GOV)**	*B* = 0.05, *SE* = 0.07, *t* = 0.69, *p* = .488	*B* = 0.06, *SE* = 0.06, *t* = 1.06, *p* = .293
**Anxiety(GOV)**	*B* = 0.03, *SE* = 0.06, *t* = 0.47, *p* = .640	*B* = -0.04, *SE* = 0.06, *t* = -0.55, *p* = .584
**Excitement(GOV)**	*B* = 0.02, *SE* = 0.05, *t* = 0.50, *p* = .619	*B* = 0.00, *SE* = 0.05, *t* = 0.05, *p* = .960
**Despair(GOV)**	*B* = -0.08, *SE* = 0.06, *t* = -1.37, *p* = .172	*B* = 0.01, *SE* = 0.06, *t* = 0.21, *p* = .834
**Anger(GOV)**	*B* = 0.00, *SE* = 0.06, *t* = -0.04, *p* = .965	*B* = 0.08, *SE* = 0.06, *t* = 1.46, *p* = .146
**Hope(GOV)**	*B* = 0.02, *SE* = 0.07, *t* = 0.29, *p* = .775	*B* = 0.8, *SE* = 0.06, *t* = 1.49, *p* = .138
**Political efficacy**	*B* = -0.08, *SE* = 0.04, *t* = -2.20, *p* = .028	*B* = -0.02, *SE* = 0.04, *t* = -0.421, *p* = .674
**Age**	*B* = -0.00, *SE* = 0.00, *t* = -0.88, *p* = .381	*B* = -0.00, *SE* = 0.00, *t* = -1.57, *p* = .117
**Sex**	*B* = -0.11, *SE* = 0.08, *t* = -1.33, *p* = .185	*B* = -0.00, *SE* = 0.09, *t* = -0.02, *p* = .981
**Religiosity**	*B* = 0.02, *SE* = 0.14, *t* = 0.15, *p* = .882	*B* = .302, *SE* = 0.10, *t* = -*3.04*, *p* = .003
**Model**	R^2 = .54; *F(29, 347)* = 14.23; *p* < .001	R^2 = .55; *F(29, 244)* = 10.38; *p* < .001

Appendix C: The Questionnaire.

Informed consent

משתתף יקר,

מטרתו של המחקר הנוכחי היא לבחון עמדות לגבי המצב החברתי ושימוש בטכנולוגיה בישראל .

במסגרת המחקר תתבקש לענות על שאלון זה בלבד. בשאלון זה תתבקש להביע את עמדותיך בנושאים הנ"ל.

המחקר נערך ע"י ד"ר בועז המאירי מבית הספר ללימודי חברה ומדיניות באוניברסיטת תל אביב
וד"ר יוסי מערבי מבית הספר אדלסון ליזמות באוניברסיטת רייכמן .

מכיוון שמדובר בעמדות אין תשובות נכונות .
הינך מתבקש להשיב ברצינות ובכנות באופן המשקף את עמדתך בצורה הטובה ביותר.
השאלון הנוכחי אורך כ-10 דקות בלבד וישמש למטרות מחקר אקדמי.

המחקר הנוכחי עשוי לקדם את הידע בנושא שימוש בטכנולוגיה .
החשיבה על נושאים אלו עשויה לסייע למשתתפים להיטיב לשקול ולהכיר את עמדותיהם בנושא.

זוהי זכותך המלאה לסרב להשיב על שאלות אשר גורמות לך אי נוחות .
במידה והנושאים העולים או השאלות הנשאלות מעוררות בך מצוקה, ניתן להפסיק את השתתפותך בשאלון.

עבור סיום המענה על השאלון תקבל פיצוי מחברת הסקרים כפי שפורסם במייל.

השאלון הוא אנונימי, לא תתבקש להזדהות בשום שלב ,
ופרטי המשתתפים ישמרו חסויים ולא יועברו לכל גורם שלישי.

בכל שאלה לגבי המחקר או זכויותיך כמשתתף בו ניתן לפנות לתמר גור
במייל tamar.gur-arie@mail.huji.ac.il :

*שאלון זה מיועד לבני כל המינים אך מנוסח בלשון זכר למטרות נוחות.

1. אני מצהיר בזאת שאני מעל גיל 18, הבנתי את כל האמור לעיל ואני מסכים להשתתף במחקר.
2. אני לא מעוניין להשתתף במחקר.

General introduction

לאחרונה פורסם דו"ח של משרד הדיגיטל הלאומי שעסק באתגרים ביישום מנגנוני קבלת החלטות מבוססי בינה מלאכותית .
עם ההתקדמות הטכנולוגית, יישום של מנגנוני קבלת החלטות המבוססים על בינה מלאכותית נהיה אפשרות ישימה וריאלית .
על כן נבקש לברר את עמדותיך ותפיסותיך לגבי שימוש במנגנוני קבלת החלטות המבוססים על בינה מלאכותית בשירות מדינת ישראל .

** Technology readiness. Based on: Lam and colleagues (2008)**

לפניך יופיעו היגדים בנושא טכנולוגיה. לגבי כל אחד מהם יש לציין עד כמה אתה מסכים עם ההיגד. ההיגדים יופיעו בסדר אקראי. אין תשובות נכונות וכל עמדה היא לגיטימית, אז אנא, ענה עליהן בכנות כדי שנוכל להבין את עמדותיך כלפי נושאים אלו באופן המיטבי.

(סולם: 1- כלל לא מסכים, 6 - לחלוטין)

1. באופן כללי, כאשר טכנולוגיה חדשה מופיעה, אתה בין הראשונים במעגל החברים שלך לרכוש אותה.
2. בדרך כלל אתה יכול להבין ולתפעל מוצרים ושירותי היי-טק חדשים ללא עזרה מאחרים.
3. זו בדיקת קריאה, עבור היגד זה יש להשיב "במידה מועטה מאד" (reading test)
4. אתה מעודכן בפיתוחים הטכנולוגיים העדכניים ביותר בתחומי העניין שלך.
5. אתה נהנה מהאתגר של הבנת ותפעול גאדג'טים טכנולוגיים.
6. הטכנולוגיה מעניקה לאנשים יותר שליטה על חיי היומיום שלהם.
7. אתה אוהב את הרעיון לעשות עסקים באמצעות מערכות מחשביות כי אתה לא מוגבל לשעות העבודה הרגילות.
8. טכנולוגיה גורמת לך להיות יותר יעיל בעבודתך.
9. טכנולוגיה מעניקה לך יותר חופש ניידות.
10. מרכזי תמיכה טכנית הם חסרי תועלת כי הם לא מסבירים דברים במונחים שאתה מבין.
11. לפעמים אתה חושב שמערכות טכנולוגיות אינן מיועדות לשימוש על ידי אנשים רגילים.
12. אין דבר כזה מדריך למוצרי היי טק או שירותים טכנולוגיים שכתוב בשפה פשוטה.
13. כשאתה מקבל תמיכה טכנית מספק של מוצר או שירות טכנולוגי, אתה לפעמים מרגיש כאילו מישהו שיודע יותר ממך מנצל אותך.
14. אתה לא חושב שזה בטיחותי למסור את פרטי כרטיס האשראי שלך באמצעות המחשב.
15. אתה לא חושב שזה בטיחותי לעשות עסקה כספית כלשהי באמצעות האינטרנט.
16. אתה חושש שאנשים אחרים יראו מידע שאתה שולח דרך האינטרנט.
17. אתה מרגיש חוסר נוחות לעשות עסקים עם בית עסק שאפשר ליצור אתו קשר רק באמצעות האינטרנט.
18. צריך שכל עסקה אלקטרונית תאושר גם בכתב באמצעות חתימה.
19. כשמשהו נעשה באופן אוטומטי, צריך לבדוק ביסודיות שהמכונה או המחשב לא עשו שום טעות.

**** Technology knowledge. Based on: Mantello and colleagues (2023)****

לפניך יופיעו היגדים נוספים. לגבי כל אחד מהם יש לציין עד כמה אתה מסכים עם ההיגד.

(סולם: 1- כלל לא מסכים, 6 - לחלוטין)

1. עד כמה אתה מכיר את הנושא של כתיבת קוד ותכנות?
2. עד כמה אתה מכיר את הנושא של מנגנוני בינה מלאכותית לקבלת החלטות?
3. עד כמה אתה מכיר את הנושא של ערים חכמות?
4. עד כמה אתה מכיר את הנושא של בינה מלאכותית?

**** Attitudes regarding AI****

לפניך יופיעו היגדים נוספים בנושא טכנולוגיה. לגבי כל אחד מהם יש לציין עד כמה אתה מסכים עם ההיגד

(סולם: 1- כלל לא מסכים, 6 - לחלוטין)

**** Attitudes regarding AI use in governance. Based on: Maaravi and Heller (2021)****

1. שימוש במנגנוני קבלת החלטות מבוססי בינה מלאכותית ככלי במסגרת ניהול המדינה הוא רעיון טוב.
2. המחשבה על שימוש במנגנוני קבלת החלטות מבוססי בינה מלאכותית ככלי במסגרת ניהול המדינה היא מחשבה לא נעימה.
3. שימוש במנגנוני קבלת החלטות מבוססי בינה מלאכותית ככלי במסגרת ניהול המדינה יסייע בקידום מדינת ישראל.

(סולם: 1- כלל לא מסכים, 6 - לחלוטין)

**** Legitimacy of using AI in governance****

1. תהליך קבלת ההחלטות של בינה מלאכותית במסגרת ניהול המדינה יהיה הוגן.
2. שימוש במנגנוני קבלת החלטות מבוססי בינה מלאכותית ככלי במסגרת ניהול המדינה מהווה סכנה גדולה.
3. שימוש במנגנוני קבלת החלטות מבוססי בינה מלאכותית ככלי במסגרת ניהול המדינה הוא יותר בטוח מקבלת החלטות של אנשים בעלי מניעים שונים.
4. שימוש במנגנוני קבלת החלטות מבוססי בינה מלאכותית לקבלת החלטות מדיניות יובילו להתנהלות מדינית שוויונית יותר.
5. לעיתים קרובות מנגנוני קבלת החלטות מבוססי בינה מלאכותית מקבלים החלטות הוגנות יותר מבני אדם.

**** Usefulness of AI in governance. Based on: Maaravi and Heller (2021)****

(סולם: 1- כלל לא מסכים, 6 - לחלוטין)

1. שימוש במנגנוני קבלת החלטות מבוססי בינה מלאכותית עשוי להאיץ תהליכים הקשורים בהתנהלות מדינית מסורבלת.
2. שימוש במנגנוני קבלת החלטות מבוססי בינה מלאכותית עשוי לשפר את יעילותם של מנגנונים מדיניים.
3. שימוש במנגנוני קבלת החלטות מבוססי בינה מלאכותית עשוי לשפר את האופן בו מדינת ישראל מנוהלת.
4. שימוש במנגנוני קבלת החלטות מבוססי בינה מלאכותית **אינו יכול** לשפר את יעילותם של מנגנונים מדיניים.
5. שימוש במנגנוני קבלת החלטות מבוססי בינה מלאכותית **אינו יכול** לשפר את האופן בו מדינת ישראל מנוהלת.
6. שימוש במנגנוני קבלת החלטות מבוססי בינה מלאכותית עשויים להקל על קבלת החלטות מדיניות טובות וניהול המדינה.

**** Attitudes regarding AI****

לפניך יופיעו היגדים נוספים בנושא טכנולוגיה. לגבי כל אחד מהם יש לציין עד כמה אתה מסכים עם ההיגד.

**** Usefulness of AI in governance. Based on: Maaravi and Heller (2021)****

(סולם: 1- כלל לא מסכים, 6 - לחלוטין)

1. שימוש במנגנוני קבלת החלטות מבוססי בינה מלאכותית עשוי להאיץ תהליכים הקשורים בהתנהלות מדינית מסורבלת.
2. שימוש במנגנוני קבלת החלטות מבוססי בינה מלאכותית עשוי לשפר את יעילותם של מנגנונים מדיניים.
3. שימוש במנגנוני קבלת החלטות מבוססי בינה מלאכותית עשוי לשפר את האופן בו מדינת ישראל מנוהלת.
4. שימוש במנגנוני קבלת החלטות מבוססי בינה מלאכותית **אינו יכול** לשפר את יעילותם של מנגנונים מדיניים.
5. שימוש במנגנוני קבלת החלטות מבוססי בינה מלאכותית **אינו יכול** לשפר את האופן בו מדינת ישראל מנוהלת.
6. שימוש במנגנוני קבלת החלטות מבוססי בינה מלאכותית עשויים להקל על קבלת החלטות מדיניות טובות וניהול המדינה.

**** Fear of personal harm due to the current government's political decisions****

(סולם: 1- כלל לא מסכים, 6 - לחלוטין)

1. האם אתה חושש שהחלטות הממשלה הנוכחית עלולות לפגוע ביכולת ההשתכרות שלך ושל משפחתך הקרובה?
2. האם אתה חושש שהחלטות הממשלה הנוכחית עלולות לפגוע בביטחון האישי שלך ושל בני משפחתך?
3. האם אתה חושש שהחלטות הממשלה הנוכחית עלולות לפגוע ברווחה שלך ושל בני משפחתך?

**** Fear of personal harm if AI is used in governance****

(סולם: 1- כלל לא מסכים, 6 - לחלוטין)

לפניך יופיעו היגדים בנושא קבלת החלטות במדינת ישראל. לגבי כל אחד מהם יש לציין עד כמה אתה מסכים עם ההיגד.

1. האם אתה חושש שהחלטות שיתקבלו באמצעות בינה מלאכותית עלולות לפגוע ביכולת ההשתכרות שלך ושל משפחתך הקרובה?
2. האם אתה חושש שהחלטות שיתקבלו באמצעות בינה מלאכותית עלולות לפגוע בביטחון האישי שלך ושל בני משפחתך?
3. האם אתה חושש שהחלטות שיתקבלו באמצעות בינה מלאכותית עלולות לפגוע ברווחה שלך ושל בני משפחתך?

**** Political efficacy****

1. כאזרח במדינה יש לך יכולת להשפיע על תהליכי קבלת ההחלטות במדינה.
2. כאזרח במדינה יש לך יכולת להשפיע על החלטות מדיניות שישפיעו על עתידך.

**** Public support (norms) regarding using AI in governance****

(סולם: 0- אף אחד, 100-כולם)

לפניך יופיעו שאלות בנושא שימוש במנגנוני קבלת החלטות שונים בישראל. יש לענות לגבי כל אחת

מהשאלות מה לדעתך הוא אחוז האנשים שמחזיקים בעמדה המתוארת.

1. מה אחוז הישראלים לדעתך התומכים בשימוש בבינה מלאכותית ככלי לקבלת החלטות מדיניות?
2. מה אחוז הישראלים לדעתך המאמינים שיש להימנע לחלוטין משימוש בבינה מלאכותית ככלי לקבלת החלטות מדיניות?
3. מה אחוז הישראלים לדעתך המאמינים שהחלטות מדיניות שהתקבלו באמצעות בינה מלאכותית היא לגיטימית?

**** Public support of the reform (norms)****

1. מה אחוז הישראלים לדעתך התומכים ברפורמות האחרונות של הממשלה?
2. מה אחוז התומכים במפלגות הקואליציה לדעתך שגם תומכים ברפורמות האחרונות של הממשלה?
3. מה אחוז התומכים במפלגות האופוזיציה לדעתך שגם תומכים ברפורמות האחרונות של הממשלה?
4. מה אחוז הישראלים לדעתך שמאמינים שיש לעצור את הצעדים שהממשלה מובילה בימים אלה?
5. מה אחוז התומכים במפלגות הקואליציה לדעתך שגם מאמינים שיש לעצור את הצעדים שהממשלה מובילה בימים אלה?
6. מה אחוז התומכים במפלגות האופוזיציה לדעתך שגם מאמינים שיש לעצור את הצעדים שהממשלה מובילה בימים אלה?

**** Warmth to various factions ****

כעת נבקש ממך לדרג את תחושותיך כלפי אנשים המשתייכים לקבוצה מסוימת באמצעות מד טמפרטורה, בין 0 ל-100. דירוג בין 0 ל-49 משמעותו תחושה קרה ושלילית כלפי אדם מהקבוצה הזו. דירוג בין 51 ל-100 משמעותו תחושה חמה וחיובית .דירוג של 50 פירושו שאינך מרגיש חמימות או קרירות כלפי אדם מהקבוצה הזו .איך היית מתאר את תחושתך כלפי הקבוצות הבאות, באופן כללי?

(סולם: 0- תחושה קרה, 100-חמימות)

**** Warmth to various factions in the government. Based on: Gidron et al., 2019****

1. הממשלה והקואליציה (בראשות בנימין נתניהו)
2. רשימת הציונות הדתית בראשות בצלאל סמוטריץ` ועוצמה יהודית
3. מפלגת הליכוד
4. ימנים
5. מפלגות האופוזיציה
6. שמאלנים

(סולם: 0- תחושה קרה, 100-חמימות)

**** Warmth towards AI. Based on: Gidron et al., 2019****

כעת נבקש ממך לדרג את תחושותיך כלפי בינה מלאכותית. איך היית מתאר את תחושתך, באופן כללי?

1. בינה מלאכותית

(סולם: 1- כלל לא מסכים, 6 - לחלוטין)

**** Emotions regarding using AI in governance****

המחשבה על בינה מלאכותית כמנגנון קבלת החלטות משמעותיות לחייך גורמת לך להרגיש:

1. פחד
2. חרדה
3. התרגשות
4. ייאוש
5. כעס
6. תקווה
7. עצב

(סולם: 1- כלל לא מסכים, 6 - לחלוטין)

**** Emotions regarding the current government ****

המחשבה על הממשלה הנוכחית כמנגנון קבלת החלטות משמעותיות לחייך גורמת לך להרגיש:

1. פחד
2. חרדה
3. התרגשות
4. ייאוש
5. כעס
6. תקווה
7. עצב

(סולם: 1- כלל לא מסכים, 6 - לחלוטין)

**** Support for the use of AI in governance decision-making. Based on: Maaravi and Heller (2021) ****

עד כמה כל אחד מההיגדים הבאים מתארים את כוונותיך במקרה שבו תעלה האפשרות לשימוש בבינה מלאכותית (שלא תוכננה על ידי צד זה או אחר במפה הפוליטית) כמנגנון קבלת החלטות מדיניות המחליף את הממשלה הנוכחית ?

1. אתמוך בשימוש בבינה מלאכותית כמנגנון קבלת החלטות מדיניות שיחליף את הכנסת (הרשות המחוקקת).
2. אתמוך בשימוש בבינה מלאכותית כמנגנון קבלת החלטות מדיניות שיחליף את הממשלה (הרשות המבצעת).
3. אתמוך בשימוש בבינה מלאכותית כמנגנון לפירוש החוקים ולבחינת הסבירות שלהם במקום הרשות השופטת.
4. אתמוך בשימוש בבינה מלאכותית כמנגנון לניהול המדינה באופן נרחב ככל הניתן.
5. אתנגד לשימוש בבינה מלאכותית כתחליף נרחב לקבלת החלטות מדיניות על ידי אנשים בעלי תפקידים.

6. אתנגד ככל הניתן לשימוש בבינה מלאכותית כתחליף לקבלת החלטות מדיניות, על מנת לוודא שכל ההחלטות יתקבלו על ידי אנשים בעלי תפקידים.
7. אתמוך בשימוש בבינה מלאכותית ככל הניתן על מנת לשנות את ההתנהלות של מדינת ישראל.
8. אתמוך בשימוש בבינה מלאכותית באופן תדיר ככל הניתן במסגרת קבלת החלטות הקשורות בניהול המדינה.

(סולם: 1- כלל לא מסכים, 6 - לחלוטין)

**** Using of AI to evaluate and shape governance decision-making ****

1. האם לדעתך כדאי להשתמש בבינה מלאכותית להערכת משרתי ציבור (ח"כים, שרים, שופטים)?
2. האם לדעתך כדאי להשתמש בבינה מלאכותית להערכת החלטות מדיניות?
3. האם לדעתך כדאי להשתמש בבינה מלאכותית לסינון מועמדים לרשויות המדינה (כנסת, ממשלה ובתי המשפט)?
4. האם אתה מודאג לגבי הגנה על האוטונומיה שלך אם תופעל בינה מלאכותית לקבלת החלטות מדיניות באופן נרחב?

**** socio-demographics ****

לקראת סיום, תישאל לגבי עמדות פוליטיות.

(סולם: 1- ימין קיצוני, 2- ימין, 3- ימין מתון, 4- מרכז, 5- שמאל מתון, 6- שמאל, 7- שמאל קיצוני)

מרבית האנשים משתמשים במונחים 'שמאל' ו'ימין' כדי להגדיר את השקפתם הפוליטית. כיצד היית מגדיר את השקפותיך הפוליטיות לפי הקטגוריות הבאות?

(סולם: 1- כלל לא מסכים, 6 - לחלוטין)

**** Identification with the group. Based on: Huddy et al., 2015****

1. עד כמה חשובה לך הזהות שלך כימני/ שמאלני?
2. עד כמה המושג ימני/ שמאלני מתאר אותך היטב?
3. כשאתה מדבר על ימנים/ שמאלנים, באיזו תדירות אתה משתמש במילה "אנחנו" לעומת המילה "הם"?
4. באיזו מידה אתה מחשיב את עצמך כימני/ כשמאלני?
5. באופן כללי אני מרגיש דומה לאנשי ימין/ שמאל אחרים.

**** Voting****

לאיזו מפלגה הצבעת בבחירות האחרונות (בנובמבר 2022)?

1. הליכוד בראשות בנימין נתניהו
2. יש עתיד בראשות יאיר לפיד
3. הציונות הדתית בראשות בצלאל סמוטריץ` ועוצמה יהודית
4. המחנה הממלכתי בראשות בני גנץ
5. ש"ס בראשות אריה דרעי
6. יהדות התורה ברשות הרב יצחק גולדקנופף

7. ישראל ביתנו בראשות אביגדור ליברמן
8. העבודה בראשות מרב מיכאלי
9. מרצ בראשות זהבה גלאון
10. הבית היהודי בראשות איילת שקד
11. רע״מ בראשות מנסור עבאס
12. חד״ש-תע״ל בראשות איימן עודה ואחמד טיבי
13. בל״ד בראשות סמי אבו שחאדה
14. אחר (פרט): ____________
15. לא הצבעתי
16. מעדיף שלא להשיב

**** News media exposure****

באיזו מידה אתה צורך חדשות (דרך האינטרנט, עיתונים, טלוויזיה, או רדיו)?

1. איני צורך חדשות כלל
2. לעתים רחוקות
3. פעם אחת לפחות במהלך השבוע
4. כמה פעמים במהלך השבוע
5. לפחות פעם אחת ביום
6. כמה פעמים בכל יום

**** Debriefing****

תודה על השתתפותך בסקר!
בסקר זה, מטרתנו הייתה לזהות קשרים בין תפיסות ועמדות שונות לגבי המצב הפוליטי והחברתי בישראל לבין תמיכה במדיניות ושימוש בבינה מלאכותית כמנגנון קבלת החלטות.
במידה ויש לך הערות או שאלות נוספות העוסקות במחקר זה, ניתן לפנות לתמר גור במייל-tamar.gur-arie@mail.huji.ac.il:

לסיום יש ללחוץ על החץ

Appendix D: The Questionnaire Translated into English.

****Informed consent****

Dear participant,

The purpose of this study is to examine attitudes regarding the social situation and use of technology in Israel.

As part of the study, all you will be asked to do is answer this questionnaire. In this questionnaire, you will be asked to express your views on the above topics.

Dr. Boaz Hameiri from the School of Social and Policy Studies at Tel Aviv University and Dr. Yossi Maaravi from the Adelson School of Entrepreneurship at Reichman University conduct the research.

Since these are opinions, there are no correct answers.

You are asked to respond seriously and honestly in a way that best reflects your position.

The current questionnaire takes only about 10 minutes and will be used for academic research purposes.

The current research may advance knowledge on the use of technology. Thinking about these topics may help participants better consider and recognize their views on the subject.

It is your full right to refuse to answer questions that make you uncomfortable. If the topics raised or the questions asked cause you distress, you can stop your participation.

For completing the questionnaire, you will receive compensation from the survey company as published in the email.

The questionnaire is anonymous, you will not be asked to identify yourself at any stage, and participants' details will be kept confidential and will not be transferred to any third party.

For any questions about the research or your rights as a participant, you can contact Tamar Gur at email: tamar.gur-arie@mail.huji.ac.il

*This questionnaire is intended for all genders but is phrased in the masculine form for convenience purposes. [authors comment: Hebrew is a gendered language in which un-gendered phrasing is uncommon]

1. I hereby declare that I am over 18 years old, I have understood all of the above, and I agree to participate in the study.
2. I do not wish to participate in the study.

****General introduction****

Recently, the National Digital Ministry published a report on challenges in implementing artificial intelligence-based decision-making mechanisms. With technological advancement, implementing decision-making mechanisms based on artificial intelligence has become a feasible and realistic option. Therefore, we would like to clarify your attitudes and perceptions regarding the use of artificial intelligence-based decision-making mechanisms in the service of the State of Israel.

**** Technology readiness. Based on: Lam and colleagues (2008)****

Statements about technology will appear before you. For each of them, please indicate how much you agree with the statement. The statements will appear in random order. There are no correct answers and every position is legitimate, so please answer them honestly so we can best understand your attitudes towards these topics.

(Scale: 1 - Strongly disagree, 6 - Strongly agree)

1. In general, you are among the first in your circle of friends to acquire new technology when it appears.
2. You can usually figure out new high-tech products and services without help from others.
3. This is a reading test, for this statement you should answer "To a very small extent" (reading test)
4. You keep up with the latest technological developments in your areas of interest.
5. You enjoy the challenge of figuring out high-tech gadgets.
6. Technology gives people more control over their daily lives.
7. You like the idea of doing business via computers because you are not limited to regular business hours.
8. Technology makes you more efficient in your occupation.
9. Technology gives you more freedom of mobility.
10. Discomfort Technical support lines are not helpful because they don't explain things in terms you understand.
11. Sometimes you think that technology systems are not designed for use by ordinary people.
12. There is no such thing as a manual for a high-tech product or service that's written in plain language.
13. When you get technical support from a provider of a high-tech product or service, you sometimes feel as if you are being taken advantage of by someone who knows more than you do.
14. Insecurity You do not consider it safe giving out a credit card number over a computer.
15. You do not consider it safe to do any kind of financial business online.
16. You worry that information you send over the Internet will be seen by other people.
17. You do not feel confident doing business with a place that can only be reached online.
18. Any business transaction you do electronically should be confirmed later with something in writing.

19. Whenever something gets automated, you need to check carefully that the machine or computer is not making mistakes.

**** Technology knowledge. Based on: Mantello and colleagues (2023)****

Additional statements will appear before you. For each of them, you need to indicate how much you agree with the statement.

(Scale: 1 - Strongly disagree, 6 - Strongly agree)

1. How familiar are you with coding and programming?
2. How familiar are you with the topic of artificial intelligence decision-making mechanisms?
3. How familiar are you with the concept of smart cities?
4. How familiar are you with the topic of artificial intelligence?

**** Attitudes regarding AI****

Additional statements about technology will appear before you. For each of them, please indicate how much you agree with the statement.

(Scale: 1 - Strongly disagree, 6 - Strongly agree)

**** Attitudes regarding AI use in governance. Based on: Maaravi and Heller (2021)****

1. The use of artificial intelligence-based decision-making mechanisms as a tool in state management is a good idea.
2. The thought of using artificial intelligence-based decision-making mechanisms as a tool in state management is an unpleasant thought.
3. The use of artificial intelligence-based decision-making mechanisms as a tool in state management will help advance the State of Israel.

**** Legitimacy of using AI in governance****

(Scale: 1- Strongly disagree, 6 - Strongly agree)

1. The decision-making process of artificial intelligence in state management will be fair.
2. The use of artificial intelligence-based decision-making mechanisms as a tool in state management poses a great danger.

3. The use of artificial intelligence-based decision-making mechanisms as a tool in state management is safer than decision-making by people with different motivations.
4. The use of artificial intelligence-based decision-making mechanisms for policy decisions will lead to more equitable state conduct.
5. Artificial intelligence-based decision-making mechanisms often make fairer decisions than humans.

** Usefulness of AI in governance. Based on: Maaravi and Heller (2021)**

(Scale: 1- Strongly disagree, 6 - Strongly agree)

1. The use of artificial intelligence-based decision-making mechanisms may accelerate processes related to cumbersome state conduct.
2. The use of artificial intelligence-based decision-making mechanisms may improve the efficiency of state mechanisms.
3. The use of artificial intelligence-based decision-making mechanisms may improve the way the State of Israel is managed.
4. The use of artificial intelligence-based decision-making mechanisms **cannot** improve the efficiency of state mechanisms.
5. The use of artificial intelligence-based decision-making mechanisms **cannot** improve the way the State of Israel is managed.
6. The use of artificial intelligence-based decision-making mechanisms may facilitate better policy decisions and state management.

** Usefulness of AI in governance. Based on: Maaravi and Heller (2021)**

** Attitudes regarding AI**

Additional statements about technology will appear before you. For each of them, you need to indicate how much you agree with the statement.

(Scale: 1- Strongly disagree, 6 - Strongly agree)

1. The use of artificial intelligence-based decision-making mechanisms may accelerate processes related to cumbersome state conduct.
2. The use of artificial intelligence-based decision-making mechanisms may improve the efficiency of state mechanisms.

3. The use of artificial intelligence-based decision-making mechanisms may improve the way the State of Israel is managed.
4. The use of artificial intelligence-based decision-making mechanisms **cannot** improve the efficiency of state mechanisms.
5. The use of artificial intelligence-based decision-making mechanisms **cannot** improve the way the State of Israel is managed.
6. The use of artificial intelligence-based decision-making mechanisms may facilitate better policy decisions and state management.

** Fear of personal harm due to the current government′s political decisions**

(Scale: 1- Strongly disagree, 6 - Strongly agree)

1. Are you concerned that the current government's decisions might harm your and your immediate family's earning capacity?
2. Are you concerned that the current government's decisions might harm your and your family's personal security?
3. Are you concerned that the current government's decisions might harm your and your family's welfare?

** Fear of personal harm if AI is used in governance**

Statements about decision-making in the State of Israel will appear before you. For each of them, please indicate how much you agree with the statement.

(Scale: 1- Strongly disagree, 6 - Strongly agree)

1. Are you concerned that decisions made through artificial intelligence might harm your and your immediate family's earning capacity?
2. Are you concerned that decisions made through artificial intelligence might harm your and your family's personal security?
3. Are you concerned that decisions made through artificial intelligence might harm your and your family's welfare?

** Political efficacy**

1. As a citizen of the country, you have the ability to influence decision-making processes in the country.
2. As a citizen of the country, you have the ability to influence policy decisions that will affect your future.

** Public support (norms) regarding using AI in governance**

Below are questions about the use of different decision-making mechanisms in Israel. For each question, please indicate what percentage of people you think hold the described position.

(Scale: 0- None, 100- Everyone)

1. What percentage of Israelis do you think support the use of artificial intelligence as a tool for policy decision-making?
2. What percentage of Israelis do you think believe that the use of artificial intelligence as a tool for policy decision-making should be completely avoided?
3. What percentage of Israelis do you think believe that policy decisions made through artificial intelligence are legitimate?

** Public support of the reform (norms)**

(Scale: 0- None, 100- Everyone)

1. What percentage of Israelis do you think support the government's recent reforms?
2. What percentage of coalition party supporters do you think also support the government's recent reforms?
3. What percentage of opposition party supporters do you think also support the government's recent reforms?
4. What percentage of Israelis do you think believe that the steps the government is currently leading should be stopped?
5. What percentage of coalition party supporters do you think also believe that the steps the government is currently leading should be stopped?
6. What percentage of opposition party supporters do you think also believe that the steps the government is currently leading should be stopped?

** Warmth to various factions **

Now, we ask you to rate your feelings towards people belonging to a certain group using a temperature gauge between 0 and 100. A rating between 0 and 49 means a cold and negative feeling towards a person from this group. A rating between 51 and 100 means a warm and positive feeling. A rating of 50 means you feel neither warmth nor coolness towards a person from this group. How would you describe your feeling towards the following groups, in general?

** Warmth to various factions in the government. Based on: Gidron et al., 2019**

(Scale: 0- Cold feeling, 100- Warmth)

1. The government and the coalition (led by Benjamin Netanyahu)
2. The Religious Zionism list led by Bezalel Smotrich and Otzma Yehudit
3. The Likud Party
4. Right-wing
5. Opposition parties
6. Left-wingers

**** Warmth towards AI. Based on: Gidron et al., 2019****

(Scale: 0- Cold feeling, 100- Warmth)

Now, we ask you to rate your feelings toward artificial intelligence. How would you describe your feelings in general?

1. Artificial Intelligence

**** Emotions regarding using AI in governance****

The thought of artificial intelligence as a mechanism for making significant decisions in your life makes you feel:

(Scale: 1 - Strongly disagree, 6 - Completely agree)

1. Fear
2. Anxiety
3. Excitement
4. Despair
5. Anger
6. Hope
7. Sadness

(Scale: 1 - Strongly disagree, 6 - Completely agree)

**** Emotions regarding the current government****

The thought of the current government as a mechanism for making significant decisions in your life makes you feel:

(Scale: 1 - Strongly disagree, 6 - Completely agree)

1. Fear
2. Anxiety
3. Excitement
4. Despair
5. Anger
6. Hope
7. Sadness

**** Support for the use of AI in governance decision-making. Based on: Maaravi and Heller (2021) **** .8

To what extent do each of the following statements describe your intentions if the possibility arises to use artificial intelligence (that was not designed by any political side) as a mechanism for policy decision-making to replace the current government?

(Scale: 1 - Strongly disagree, 6 - Completely agree)

1. I will support the use of artificial intelligence as a mechanism for policy decision-making to replace the Knesset (the legislative branch).
2. I will support the use of artificial intelligence as a mechanism for policy decision-making to replace the government (the executive branch).
3. I will support the use of artificial intelligence as a mechanism for interpreting laws and examining their reasonableness instead of the judiciary.
4. I will support the use of artificial intelligence to manage the state as broadly as possible.
5. I will oppose the widespread use of artificial intelligence as a substitute for policy decision-making by individuals holding positions.
6. I will oppose the use of artificial intelligence as much as possible as a substitute for policy decision-making to ensure that all decisions are made by individuals holding positions.
7. I will support the use of artificial intelligence as much as possible to change the conduct of the State of Israel.
8. I will support the frequent use of artificial intelligence in decision-making related to the management of the state.

**** Using AI to evaluate and shape governance decision-making****

(Scale: 1 - Strongly disagree, 6 - Completely agree)

1. Do you think it is advisable to use artificial intelligence to evaluate public officials (Knesset members, ministers, judges)?
2. Do you think it is advisable to use artificial intelligence to evaluate policy decisions?
3. Do you think it is advisable to use artificial intelligence to screen candidates for state authorities (Knesset, government, and judiciary)?
4. Are you concerned about protecting your autonomy if artificial intelligence is widely used for policy decision-making?

**** socio-demographics ****

Before we finish, you will be asked about political positions.

(Scale: 1 - Far-right, 2 - Right, 3 - Moderate right, 4 - Center, 5 - Moderate left, 6 - Left, 7 - Far-left)

Most people use the terms "left" and "right" to define their political views. How would you define your political views based on the following categories?

**** Identification with the group. Based on: Huddy et al., 2015****

(Scale: 1 - Strongly disagree, 6 - Completely agree)

1. How important is your identity as right-wing/left-wing to you?
2. How well does the term right-wing/left-wing describe you?
3. When you talk about right-wing/left-wing people, how often do you use the word "we" versus "they"?
4. To what extent do you consider yourself right-wing/left-wing?
5. In general, I feel similar to other right-wing/left-wing people.

**** Voting****

Which party did you vote for in the last election (in November 2022)?

1. Likud, led by Benjamin Netanyahu
2. Yesh Atid, led by Yair Lapid
3. Religious Zionism, led by Bezalel Smotrich and Otzma Yehudit
4. National Unity, led by Benny Gantz
5. Shas, led by Aryeh Deri
6. United Torah Judaism, led by Rabbi Yitzhak Goldknopf
7. Yisrael Beiteinu, led by Avigdor Lieberman
8. Labor, led by Merav Michaeli
9. Meretz, led by Zehava Galon
10. Jewish Home, led by Ayelet Shaked
11. Ra'am, led by Mansour Abbas
12. Hadash-Ta'al, led by Ayman Odeh and Ahmad Tibi
13. Balad, led by Sami Abu Shehadeh
14. Other (please specify): ___________
15. I did not vote
16. Prefer not to answer

**** News media exposure****

To what extent do you consume news (through the internet, newspapers, television, or radio)?

1. I do not consume news at all
2. Rarely
3. At least once during the week
4. Several times during the week
5. At least once a day
6. Several times a day

**** Debriefing****

Thank you for participating in the survey!
In this survey, our goal was to identify connections between perceptions and attitudes regarding the political and social situation in Israel and support for policies and the use of artificial intelligence as a decision-making mechanism.
If you have any further comments or questions regarding this research, you can contact Tamar Gur by email: tamar.gur-arie@mail.huji.ac.il

To finish, click the arrow.

References

Israel Central Bureau Of Statistics (2021). Population - Statistical Abstract of Israel 2021 - No.72. *Israel Central Bureau Of Statistics website*. Retrieved: on November 30th 2023: https://www.cbs.gov.il/he/publications/Pages/2021/%D7%90%D7%95%D7%9B%D7%9C%D7%95%D7%A1%D7%99%D7%99%D7%94-%D7%A9%D7%A0%D7%AA%D7%95%D7%9F-%D7%A1%D7%98%D7%98%D7%99%D7%A1%D7%98%D7%99-%D7%9C%D7%99%D7%A9%D7%A8%D7%90%D7%9C-2021-%D7%9E%D7%A1%D7%A4%D7%A8-72.aspx

Israel Central Bureau Of Statistics (2020). Population, by population group, religion, sex and age. *Israel Central Bureau Of Statistics website*. Retrieved: November 30th 2023: https://www.cbs.gov.il/he/publications/doclib/2020/2.shnatonpopulation/st02_03.pdf

Israel Democracy Institute (2022). 2022 Elections. *Israel Democracy Institute website*. Retrieved: November 30th 2023: https://www.idi.org.il/policy/parties-and-elections/elections/2022-1/
